# Clinicopathological characterization of enteric glia in colorectal cancer: Insights from a population-based cohort

**DOI:** 10.1093/jnen/nlaf067

**Published:** 2025-06-28

**Authors:** Meike S Thijssen, Maartje Massen, Jaleesa R M van der Meer, Marion J Gijbels, Iryna V Samarska, Manon van Engeland, Matty P Weijenberg, Piet A van den Brandt, Kim M Smits, Werend Boesmans, Veerle Melotte

**Affiliations:** Department of Pathology, GROW—Research Institute for Oncology and Reproduction, Maastricht University Medical Center, Maastricht, The Netherlands; Biomedical Research Institute (BIOMED), Hasselt University, Hasselt, Belgium; Department of Pathology, GROW—Research Institute for Oncology and Reproduction, Maastricht University Medical Center, Maastricht, The Netherlands; Department of Pathology, GROW—Research Institute for Oncology and Reproduction, Maastricht University Medical Center, Maastricht, The Netherlands; Department of Pathology, GROW—Research Institute for Oncology and Reproduction, Maastricht University Medical Center, Maastricht, The Netherlands; Department of Medical Biochemistry, Experimental Vascular Biology, Amsterdam Cardiovascular Biology, Amsterdam Cardiovascular Sciences, Amsterdam Infection and Immunity, Amsterdam UMC, Amsterdam, The Netherlands; Department of Pathology, GROW—Research Institute for Oncology and Reproduction, Maastricht University Medical Center, Maastricht, The Netherlands; Department of Pathology, GROW—Research Institute for Oncology and Reproduction, Maastricht University Medical Center, Maastricht, The Netherlands; Department of Epidemiology, GROW—Research Institute for Oncology and Reproduction, Maastricht University, Maastricht, The Netherlands; Department of Epidemiology, GROW—Research Institute for Oncology and Reproduction, Maastricht University, Maastricht, The Netherlands; Department of Pathology, GROW—Research Institute for Oncology and Reproduction, Maastricht University Medical Center, Maastricht, The Netherlands; Department of Pathology, GROW—Research Institute for Oncology and Reproduction, Maastricht University Medical Center, Maastricht, The Netherlands; Biomedical Research Institute (BIOMED), Hasselt University, Hasselt, Belgium; Department of Pathology, GROW—Research Institute for Oncology and Reproduction, Maastricht University Medical Center, Maastricht, The Netherlands; Department of Clinical Genetics, Erasmus University Medical Center, Rotterdam, The Netherlands

**Keywords:** clinicopathological characterization, colorectal tumorigenesis, glial cells, glial fibrillary acidic protein, tumor microenvironment

## Abstract

Enteric glia contribute to the regulation of mucosal homeostasis and intestinal immunity. Enteric glia dysfunction is linked to various gastrointestinal disorders. We aimed to characterize the phenotype of enteric glia in colorectal cancer (CRC) and examine their association with CRC patient characteristics. Healthy, adenoma, and tumor tissues from CRC patients were immunohistochemically stained for the glial markers S100B and glial fibrillary acidic protein (GFAP). GFAP-positive enteric glia were identified within carcinoma tissue stroma but were absent in normal mucosa or adenoma tissue from the same patients. S100B staining was detected in all sample types. Two CRC patient cohorts (*n* = 447 and *n* = 324) were analyzed for GFAP staining and to assess association of GFAP immunoreactivity with patient characteristics. This indicated that GFAP-positive cells might be associated with tumor localization and median survival. High-density GFAP staining was associated with improved survival in the study cohort (HR = 0.56; *P* = 0.030), but not the validation cohort (HR = 0.85; *P* = 0.606). These findings suggest that CRC induces GFAP expression in enteric glia. While prognostic value of GFAP could not be confirmed, future studies are needed to elucidate the role of enteric glia in CRC prognosis and progression.

## INTRODUCTION

Colorectal cancer (CRC) is a complex and heterogeneous disease. This is partly due to the prominent role of the tumor microenvironment (TME), which consists of a large variety of cells surrounding the tumor including fibroblasts, immune cells, endothelial cells, and extracellular matrix components.^1–3^ Over the last years, the role of (enteric) neurons and glial cells as members of the colorectal cancer microenvironment has been considered.^4–8^ This is not surprising as the gut is a highly innervated organ and neural cells are one of the most important gatekeepers of gut function and homeostasis. In addition to afferent and efferent branches of extrinsic origin, an extensive intrinsic network of nerve cells and glia, the enteric nervous system, is located within the gut wall.^9^ While the cell bodies of enteric neurons are confined within ganglionated plexus layers, enteric glia can also be found in the other layers of the gut wall including the lamina propria.^10^ Different types of enteric glia are classified based on their morphology and location.^11^^,^^12^ They are active players in enteric neurotransmission, influence the development and maturation of mucosal epithelial cells and take part in intestinal immune responses.^13–16^ Furthermore, enteric glia exhibit a high degree of plasticity, adapting quickly to changes in their environment.^10^^,^^17^ For instance, it has been shown that enteric glial cells can take on a so-called “reactive” phenotype in inflammatory conditions.^18–21^ Their functional importance together with their high level of phenotypic plasticity and reactivity in response to disease puts enteric glia in a prime position to interact with tumor cells and contribute to colorectal carcinogenesis. Previous studies showed the presence of glial cells in CRC tumor stroma and an increase of glia when compared to normal colonic mucosal tissue.^5^^,^^22^^,^^23^ In addition, a higher enteric glial cell density was observed in well-differentiated tumors compared to moderately and poorly differentiated tumors in, albeit relatively small, CRC patient cohorts.^24–26^

A limited number of in vitro and in vivo studies have shown an effect of enteric glia on CRC development and progression. Enteric glial cells increased the number and size of tumorspheres in vitro via paracrine signaling.^22^ In vivo experiments have confirmed a tumor-promoting role for enteric glial cells in CRC development by showing an increase in tumor size in mice when CRC cells were injected together with enteric glia,^22^ and a reduced tumor burden in mice with glial fibrillary acidic protein (GFAP) promotor-driven depletion of enteric glia.^27^ Recently, enteric glia were shown to communicate with the immune TME, specifically tumor-associated macrophages, in CRC both in vitro and in vivo.^28^ In a positive feedback system, monocytes and macrophages within the TME produced IL-1, which stimulated enteric glia to become reactive and pro-tumorigenic. In turn, these reactive glial cells increased IL-6 production promoting the differentiation of monocytes toward SPP1+ tumor-associated macrophages.

Building on the studies mentioned above, it is evident that enteric glia play a significant role in the colorectal TME. However, their phenotype in the human cancerous setting remains unclear and their possible impact on CRC patient characteristics and prognosis has not yet been investigated. In this study, we characterized enteric glia within the CRC microenvironment and examined the association of the enteric glial cell marker GFAP with CRC patient characteristics in two large population-based patient cohorts.

## METHODS

### Study populations and tissue samples

For patient-matched normal, adenoma [tubulo(villous) with low grade dysplasia] and carcinoma tissue, formalin-fixed, paraffin-embedded (FFPE) colorectal cancer tissues (*n* = 90) were retrospectively retrieved from the tissue archive of the Department of Pathology of Maastricht University Medical Center.^29^ This cohort was set up between 1995 and 2003 and included patients >50 years of age at time of CRC diagnosis. From these patients, normal colon mucosa (*n* = 79) and adenoma (*n* = 62) were also retrieved when available. We used a randomly selected subset of 10 patients for which carcinoma, adenoma, and normal colon mucosa tissue samples were available.

For the population-based series of CRC patients, formalin fixed, paraffin-embedded tumor tissue samples from the Netherlands Cohort Study on diet and cancer (NLCS) were used. The NLSC has been described in more detail previously.^30^ In brief, the NLCS is a prospective patient cohort initiated in 1986 with the inclusion of 120 852 healthy individuals aged 55-69 years old. At baseline all participants completed a self-administered questionnaire on diet, family history of cancer and other risk factors.^30^ Follow-up for cancer incidence was established by annual record linkage with the Netherlands Cancer Registry (NCR) and the nationwide Dutch Pathology Registry (PALGA).^31^^,^^32^ Completeness of cancer incidence was estimated >96%.^33^ Follow-up for vital status of CRC cases was carried out through linkage to the Central Bureau of Genealogy and the municipal population registries until December 31, 2012. Cause of death was retrieved from Statistics Netherlands. FFPE tissue blocks of colon tumors from NLCS patients were collected at two moments. First, tumor material was collected for 734 CRC patients identified in the NLCS from 1989 to 1994, excluding the first 2.3 years of follow-up.^34^ For our study, tissue of 723 patients was available of which we used a randomly selected subset as study cohort (*n* = 447; Table 1). Second, tissue blocks of CRC patients diagnosed in the NLCS until 01-01-2007, were collected (*n* = 3021) as part of the Rainbow-Tissue Microarray (TMA) project.^35^ After pathological review, tissue blocks of 2694 patients was available for further analysis. As in-cohort validation, a random subset (*n* = 315) of this second collection period was used (Table 1). Information regarding tumor characteristics (eg, tumor grade, location, staging, differentiation grade) was made available through the Netherlands Cancer Registry.^34^

**Table 1.** Clinicopathological characteristics of the study cohort and in-cohort validation set.

Patient demographics	Study cohort, *n* (%)	In-cohort validation, *n* (%)
Total	447	315
**Sex**		
Male	258 (57.7)	168 (53.3)
Female	189 (42.3)	147 (46.7)
		***P* = 0.230**
**Age at diagnosis (years)**		
Mean Age (± SD)	67.9 (4.3)	74.3 (6.0)
		***P* < 0.001**
**Cancer stage (TNM)**		
Stage I	120 (30.3)	61 (19.9)
Stage II	141 (33.7)	119 (38.8)
Stage III	100 (12.7)	74 (24.1)
Stage IV	68 (23.3)	53 (17.3)
		***P* = 0.078**
**Localization**		
Colon	283 (64.0)	223 (78.8)
Rectosigmoid	56 (12.7)	30 (9.5)
Rectum	103 (23.3)	62 (19.7)
		***P* = 0.138**
**Differentiation grade**		
Undifferentiated	6 (1.5)	2 (0.7)
Poor	60 (15.3)	53 (18.2)
Moderate	278 (70.7)	208 (71.5)
Well	49 (12.5)	28 (9.6)
		***P* = 0.376**
**CRC death**		
Yes	183 (41.1)	141 (45.5)
No	262 (58.9)	169 (54.5)
		***P* = 0.234**
**Survival (years)**		
Median (± SD)	8.0 (5.5)	4.3 (5.9)
		***P* < 0.001**

Abbreviations: CRC = colorectal cancer, *n* = number of patients, SD = standard deviation, TNM = tumor-lymph node-metastasis.

### Immunohistochemistry

Paraffin sections (3-4 µm) of colorectal tumor tissue were selected for immunohistochemical staining for GFAP or S100B. The patient cohort with matched normal, adenoma and carcinoma tissue was stained manually. Shortly, endogenous peroxidase activity was blocked by 0.3% hydrogen peroxide in methanol for 5 min, tissues were incubated with primary antibody (polyclonal rabbit anti-S100B 1:600, Proteintech; polyclonal rabbit anti-GFAP 1:1000, DAKO) for 45 min and subsequently with secondary antibody (Brightvision anti-mouse/rabbit IgG HRP) for 20 min. For the NLCS patient cohorts, staining was carried out using the DAKO autostainer link 48. First, tissue sections were incubated in EnVision FLEX target retrieval solution (pH 9.0; DAKO) at 97 degrees for 10 min. Then, endogenous peroxidase activity was blocked with peroxidase-blocking solution (DAKO) for 5 min. Sections were incubated with the primary antibody polyclonal rabbit anti-GFAP (ready-to-use; DAKO) for 20 min and with the secondary antibody Poly-HRP IgG conjugate (DAKO) for 20 min. For all slides, visualization was done by 3,3’-diaminobenzidine substrates (DAB, DAKO) as a chromogen. Counterstaining was performed by hematoxylin and slides were mounted with xylene in the Sakura Tissue-Tek Prisma & Film.

### Histological slide imaging

**Figure 1.**
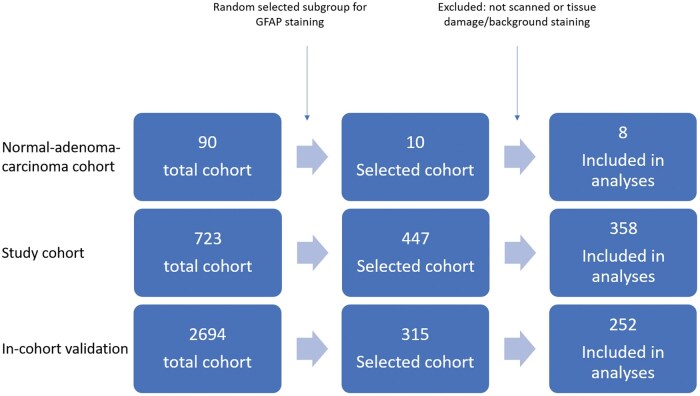
Flowchart showing sample numbers within the normal-adenoma-carcinoma cohort, the study population and in-cohort validation population. A randomly selected subset of 10 patients was selected from the tissue archive for which carcinoma, adenoma and normal colon mucosa tissue was available. For the study cohort, a random selection of 447 patient slides was collected from the total cohort (*n* = 723). The in-cohort validation cohort contained 315 selected patient slides from the total cohort (*n* = 2694). Some slides were excluded for further analyses due to damage to the slide, failure in the scanning process or too much background staining.

For the patient-matched normal-adenoma-carcinoma cohort, slides from 8 patients were successfully stained and included for further analysis, whereas the NLCS study cohort included 358 slides and the in-cohort validation subset included 252 slides for further analysis (Figure 1). In case of heavy damage, lack of tumor material on slides or too much nonspecific staining, slides were excluded. The included slides were scanned with the 3D HISTECH PANNORAMIC 1000 scanner using 20x magnification. The digitized slides were viewed using the CaseViewer tool (version 2.4.0.119028). All scanned slides could be assessed with a magnification from 0.2× to 63× using the digital zoom option in the CaseViewer application.

### Histological assessment

For histological assessment, the tumor tissue including intratumoral stroma was manually outlined in the CaseViewer application, and further addressed as the tumor area. Tumor areas that showed significant damage or folds were excluded. Successful plexus staining was used as internal positive control. All tumor areas were assessed and classified for staining density of GFAP by two independent observers (M.S.T. & M.M.). Three distinct values for staining density could be applied: negative staining, low-density staining (few single cells or one cluster) or high-density staining (many single cells and/or multiple clusters). In case of disagreement between the two observers, cases were re-examined and discussed until consensus was reached.

### Data analysis

For the population-based series, descriptive statistics and frequency distributions were calculated for clinical characteristics [age at diagnosis, sex, tumor-lymph node-metastasis (TNM) stage, tumor localization, and differentiation grade]. Differences between staining subgroups (ie, negative, low-density, and high-density staining) were evaluated using Pearson chi-square test for categorical variables and *t*-tests or Kruskal-Wallis for continuous variables. CRC cause-specific survival was defined as time from cancer diagnosis until CRC-related death or end of follow-up; deaths within two weeks after surgery were excluded for all analyses. Univariable survival analyses were performed using Kaplan-Meier and log rank tests. Hazard ratios (HR) and corresponding 95% confidence intervals (CI) were assessed using Cox proportional hazard models adjusted for *a priori* selected potential confounders and known prognostic factors (ie, TNM stage, localization, differentiation grade, and age at diagnosis). The proportional hazards assumption was tested using the scaled Schoenfeld residuals,^36^ by evaluating -log-log transformed survival curves. Statistical analyses were performed using STATA17.0.

## RESULTS

### GFAP expression is upregulated in enteric glial cells in a CRC microenvironment

**Figure 2.**
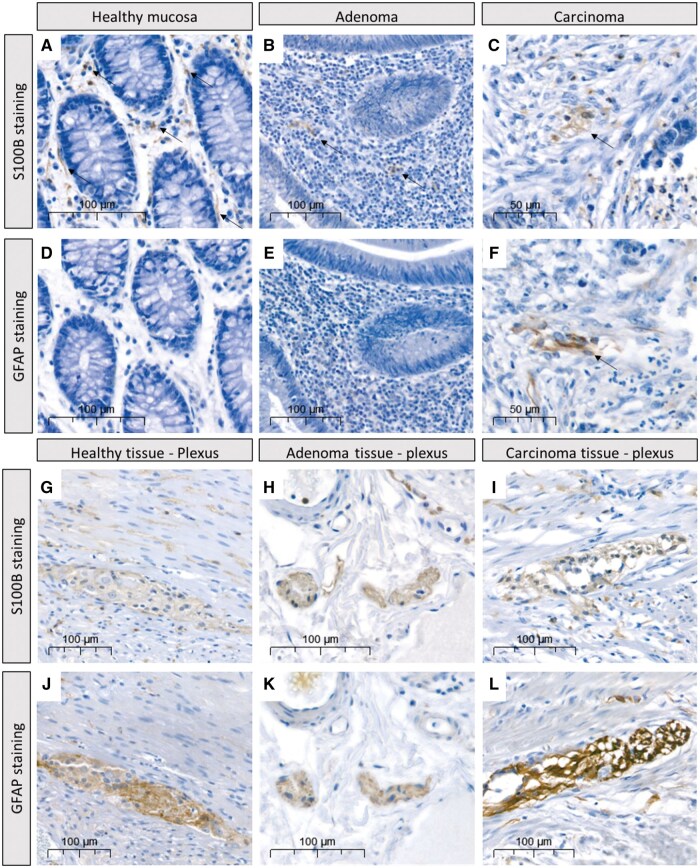
Representative images of S100B and GFAP staining within mucosal, adenoma and carcinoma tissue of the same patient, and corresponding plexus staining. (A) Normal mucosa, (B) adenoma tissue, and (C) carcinoma tissue from the same patient show S100B staining. (D) Normal mucosa and (E) adenoma tissue do not show GFAP staining, while (F) GFAP staining is present in carcinoma tissue from the same patient. (G-L) For all tissues, ganglia within the plexus layers stain positive for S100B (G-I), and GFAP (J-L) and thereby serve as positive controls.

To investigate the presence and marker expression of enteric glia, we evaluated S100B and GFAP expression in human samples of adjacent normal mucosa, adenoma, and carcinoma tissue from the same CRC patient. Given the dynamic regulation of GFAP expression levels,^11^ and its use as marker for enteric gliosis,^14^^,^^20^ we used immunoreactivity for S100B to locate enteric glia within the tissue samples.^37^ Both S100B and GFAP were shown to be extensively expressed in the plexus layers of all tissue samples (Figure 2G-L). Furthermore, S100B staining was found in normal mucosa (Figure 2A), adenoma (Figure 2B), and carcinoma tissue (Figure 2C) of all patients, confirming the presence of enteric glial cells. However, GFAP staining was absent in normal mucosal tissue (0 of 8 patients; Figure 2D), rarely found in adenoma tissue (staining in 1 of 8 patients; Figure 2E), but abundantly present in carcinoma tissue (staining in 6 of 8 patients, Figure 2F). These data indicate that enteric glia upregulate GFAP in human CRC tumors compared to mucosal enteric glia in non-neoplastic tissue samples.

### Different densities of GFAP-positive enteric glia are found within intratumoral CRC stroma

**Figure 3.**
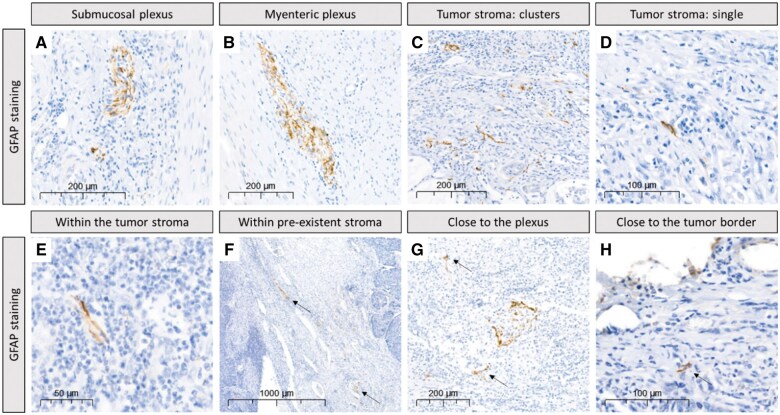
Representative images of GFAP staining within the plexus layers and the tumor stroma. (A) Submucosal and (B) myenteric ganglia stain positive for GFAP and thereby serve as positive control on the slides. (C-H) Positive staining for GFAP was also observed in the tumor stroma. Both clusters of multiple GFAP-positive cells (C) and single GFAP-positive cells (D) were found in intratumoral stroma. GFAP-positive glial cells were observed in varying locations throughout the tumor, such as (E) within the tumor stroma, (F), within peritumoral pre-existent stroma, (G) close to the plexus, and (H) close to the tumor border. Arrows point to GFAP-positive cells that correspond with the described locations.

To investigate whether GFAP expression is associated with patient characteristics, GFAP staining was successfully performed on a study cohort of 358 human patient samples and an in-cohort validation of 252 human patient samples (Figure 3A and B). GFAP expression in enteric glia in CRC tumor tissue showed large variation between patients and was found in varying locations within the tumors, ie, within the tumor stroma (Figure 3E), within peritumoral pre-existent stroma (Figure 3F), within intratumoral stroma in proximity to the plexus (Figure 3G), and toward the edge of the tumor distant from the plexus (Figure 3H). GFAP-positive cells within ganglia of the plexus could also be found in tumor areas but were not included in our analysis. GFAP immunoreactivity within intratumoral stroma could be detected both from clusters of cells and from single cells (Figure 3C and D). Therefore, GFAP-positive samples were categorized into two groups for further analysis, ie, low-density staining (few single cells or one cluster) and high-density staining (many single cells and/or multiple clusters).

### GFAP expression is not associated with specific patient or tumor characteristics

Overall, more GFAP-positive tumors were found (study cohort: *n* = 257, 71.8%; in-cohort validation: *n* = 181, 71.8%) compared to GFAP-negative tumors (study cohort: *n* = 101, 28.2%; in-cohort validation: *n* = 71, 28.2%) (Table 2). The low-density GFAP staining group was overall larger (study cohort: *n* = 173, 48.3%; in-cohort validation: *n* = 129, 51.2%) than the high-density staining group (study cohort: *n* = 84, 23.5%; in-cohort validation: *n* = 52, 20.6%). In both cohorts, male patients represented the majority of the population but this was independent of GFAP staining group (*P* = 0.711 in the study cohort vs *P* = 0.596 in the in-cohort validation). In the study population, CRC stage I tumors were more often GFAP-negative (*n* = 34, 35.0%), while low-density and high-density GFAP staining was mainly found within CRC stage ll (*n* = 70, 41.4% and *n* = 26, 32.1% respectively; *P* = 0.068). In the in-cohort validation, no differences were observed for TNM stage distribution in the different staining groups (*P* = 0.869). Negative staining appeared to be more frequent in tumors located in the colon compared to low-density and high-density staining in the study cohort (*n* = 72, 73.5% vs *n* = 112, 65.1% and *n* = 53, 63.9%). This was also observed in the in-cohort validation [*n* = 56, 78.9% vs *n* = 92, 71.3% (low-density) and *n* = 35, 67.3% (high-density)], but these differences were not statistically significant (*P* = 0.688 in the study cohort and *P* = 0.257 in the in-cohort validation). Similarly, no significant differences were observed between the staining groups for CRC mortality or differentiation grade; most tumors were moderately differentiated in both populations (Table 2).

**Table 2.** Associations between GFAP expression and clinicopathological features: sex, TNM-stage, localization, differentiation grade, CRC death and median survival.

Patient variables	GFAP staining study cohort			GFAP staining in-cohort validation		
	Negative staining *n* (%)	Low-density staining, *n* (%)	High-density staining, *n* (%)	Negative staining *n* (%)	Low-density staining, *n* (%)	High-density staining, *n* (%)
**Total**	101 (28.2)	173 (48.3)	84 (23.5)	71 (28.2)	129 (51.2)	52 (20.6)
**Mean age**						
Mean (± SD)	68.0 (4.3)	67.8 (4.3)	68.7 (4.1)	74.4 (6.2)	74.7 (6.1)	73.7 (6.1)
			***P* = 0.294**			***P* = 0.607**
**Sex**						
Male	55 (54.4)	103 (59.5)	48 (57.1)	36 (50.7)	68 (52.7)	31 (59.6)
Female	46 (45.6)	70 (40.5)	36 (42.9)	35 (49.3)	61 (47.3)	21 (40.4)
			***P* = 0.711**			***P* = 0.596**
**Cancer stage (TNM)**						
Stage I	34 (35.0)	38 (22.5)	22 (27.2)	13 (18.3)	26 (20.8)	9 (17.7)
Stage II	25 (25.8)	70 (41.4)	26 (32.1)	26 (36.6)	50 (40.0)	22 (43.1)
Stage III	23 (23.7)	41 (24.3)	16 (19.7)	16 (22.5)	31 (24.8)	11 (21.6)
Stage IV	15 (15.5)	20 (11.8)	17 (21.0)	16 (22.5)	18 (14.4)	9 (17.7)
			***P* = 0.068**			***P* = 0.869**
**Localization**						
Colon	72 (73.5)	112 (65.1)	53 (63.9)	56 (78.9)	92 (71.3)	35 (67.3)
Rectosigmoid	9 (9.2)	19 (11.1)	13 (15.7)	7 (9.9)	12 (9.3)	3 (5.8)
Rectum	17 (17.4)	41 (23.8)	17 (20.4)	8 (11.3)	25 (19.4)	14 (26.9)
			***P* = 0.688**			***P* = 0.257**
**Differentiation grade**						
Undifferentiated	0 (0)	4 (2.6)	5 (6.3)	1 (1.6)	1 (0.8)	0 (0)
Poor	12 (13.8)	27 (17.5)	14 (17.7)	11 (17.2)	26 (21.1)	7 (14.9)
Moderate	61 (70.1)	107 (69.5)	59 (74.7)	46 (71.9)	87 (70.7)	35 (74.5)
Well	14 (16.1)	16 (10.4)	5 (16.3)	6 (9.4)	9 (7.3)	5 (10.6)
			***P* = 0.329**			***P* = 0.902**
**CRC death**						
Yes	41 (40.6)	69 (39.9)	30 (36.6)	28 (40.6)	55 (43.0)	24 (48.0)
No	60 (59.4)	104 (60.1)	52 (63.4)	41 (59.4)	73 (57.0)	26 (52.0)
			***P* = 0.840**			***P* = 0.718**
**Median survival**						
Median (± SD)	6.8 (5.5)	8.9 (5.5)	11.4 (5.7)	4.1 (6.5)	4.8 (5.9)	6.4 (5.6)
			***P* = 0.183**			***P* = 0.957**

Abbreviations: GFAP = glial fibrillary acidic protein, CRC = colorectal cancer, *n* = number of patients, SD = standard deviation, TNM = tumor-lymph node-metastasis.

Although not statistically significant, the lowest median survival time was found for patients with a GFAP-negative tumor in the study cohort (6.8 years for GFAP-negative tumors vs 8.9 in the low-density group), but this was not observed in the in-cohort validation (4.1 vs 4.8 years). In contrast, patients with a high-density staining had the highest median survival in both cohorts (11.4 years in the study cohort and 6.4 in the in-cohort validation). Strikingly, in both populations, patients for which GFAP staining failed had the lowest median survival (4.9 ± 5.1 years in the study cohort and 2.8 ± 5.5 for the in-cohort validation) (Table S1).

**Figure 4.**
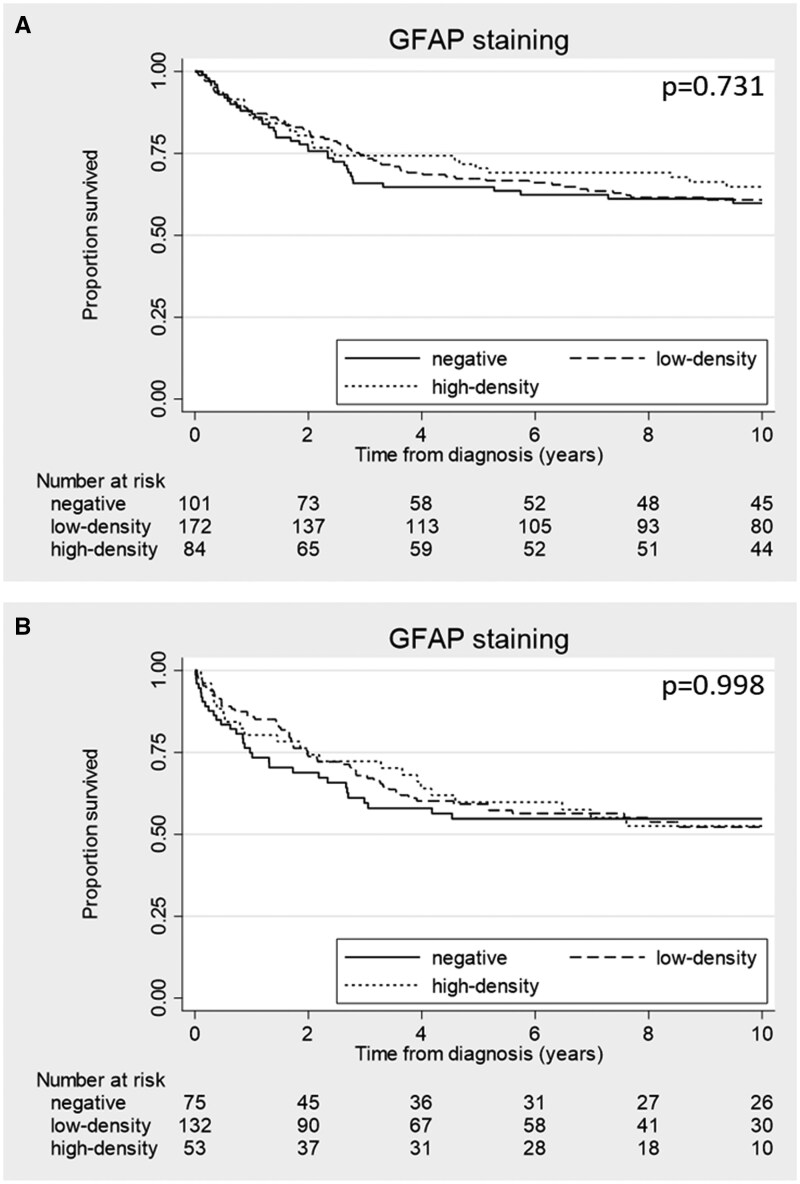
Kaplan-Meier curves of the 10-year cancer specific survival curve for GFAP staining within the CRC patient study cohort (A) and in-cohort validation cohort (B). The straight lines represent GFAP-negative samples’ the striped lines represent low-density GFAP samples and the dotted lines represent high-density GFAP samples. (A) High-density GFAP staining is associated with better survival compared to GFAP-negative staining (HR = 0.56; 95%-CI 0.33-0.95, *P* = 0.030) in the study cohort. (B) No significant difference in survival is associated with GFAP staining is observed for the in-cohort validation (HR = 0.93; 95%-CI 0.55-1.59, *P* = 0.802).

Kaplan-Meier analyses did not indicate a survival difference for the GFAP staining groups (*P* = 0.731) in the study cohort (Figure 4A). However, after adjusting for age at diagnosis, sex, TNM-stage, localization and differentiation grade, the GFAP high-density staining group showed a significantly better survival rate compared to the GFAP-negative group (HR = 0.56; 95% CI 0.33-0.94; *P* = 0.029). A similar trend was seen in the low-density staining group although this was not statistically significant (HR = 0.83; 95% CI 0.53-1.29; *P* = 0.398). The in-cohort validation showed no significant survival differences in the Kaplan Meier analyses (*P* = 0.998) (Figure 4B) and the survival benefit in the high-density GFAP staining group could not be validated in multivariate analyses (HR = 0.92; 95%-CI 0.62-1.66, *P* = 0.786). Similarly, the trend toward better survival in the low-density staining group could not be validated (HR = 1.02; 95%-CI 0.62-1.66; *P* = 0.949).

Together, these data suggest that GFAP-positive enteric glia might be associated with tumor localization and median survival but not with other CRC patient characteristics. Moreover, GFAP seemed to harbor prognostic potential in the study cohort but this result is inconsistent with the in-cohort validation.

## DISCUSSION

In this study, we explored the effect of a CRC environment on enteric glia and investigated the distribution of the immunohistochemical marker GFAP in the tumor and its association with clinicopathological characteristics in a large population-based CRC cohort. We show that enteric glial cells upregulate GFAP in a CRC environment. The density of GFAP staining in the tumor stroma is not associated with CRC patient characteristics, such as tumor stage, differentiation grade and mortality, but might be associated with tumor localization and median survival, however not significantly. The prognostic potential of GFAP remains unclear as survival analysis results were inconsistent between the two cohorts.

To date, human studies focusing on enteric glia in CRC are limited; this is the first large population-based study to investigate a possible association between enteric glial cells and CRC patient characteristics. The presence of enteric glia using S100B and GFAP staining in CRC tumor tissue and an increase compared to normal human colon tissue was previously shown by other groups in a limited number of human samples.^5^^,^^22^^,^^23^ Here, using a large population-based cohort of CRC patients, we confirmed that GFAP-positive enteric glial cells are present within intratumoral stroma. Moreover, we observed that GFAP expression, one of the hallmarks of enteric gliosis,^14^^,^^20^ was absent from glia in normal mucosa and rarely found in adenoma tissue.

Within the human tumor tissue, GFAP immunoreactive cells were observed in different locations. Besides their presence in intratumoral stroma, GFAP-positive cells were also observed in peritumoral pre-existent stroma. This raises the question as to whether these cells were pushed there passively together with the stroma during tumor formation or actively migrated toward the tumor, possibly attracted by tumor-derived signals that change the stroma into a favorable environment. Both of these mechanisms have been described.^38^^,^^39^ Furthermore, enteric glia were observed within the tumor close to plexus structures but also deep into the tumor or at the tumor border. Since we observed S100B-expressing enteric glia in normal mucosa, adenoma and carcinoma tissue, we hypothesize that these cells represent mucosal enteric glia that upregulate GFAP, rather than cells that migrated centripetally from outer plexus layers into the tumor.

Previously, population-based studies with small CRC patient cohorts showed the highest GFAP-positive enteric glial cell density in well-differentiated tumors and decreased density in moderately to poorly differentiated tumors.^24^^,^^25^ Prognostic information however was not available for these small cohorts. Also, enrichment of enteric glia in the tumor was associated with metastasis and poor prognosis of CRC patients in another small cohort.^23^ In contrast to the previously published studies,^24^^,^^25^ no association was observed between GFAP staining and differentiation grade in our large patient cohorts. Moreover, in our study cohort, GFAP immunohistochemistry suggested a better survival for patients with high-density staining independent of known established prognostic factors such as TNM stage and tumor differentiation. However, our in-cohort validation could not reproduce the survival results observed in the study cohort, although median survival was highest for patients with a high-density GFAP staining in that cohort as well. These differences in outcome for our two cohorts could originate from the dissimilarities between the populations, such as the higher age at diagnosis, the different stage and location distributions and the differences in median survival. Furthermore, longer storage of the FFPE slides from the study cohort compared to the in-cohort validation could have affected antigenicity.^40^ However, the percentage of negative staining was the same in both cohorts and plexus structures in the tissues were used as internal positive controls to ensure staining success.

It is important to consider that we are using GFAP expression to mark enteric glial cells, which is highly time- and location dependent and sensitive to environmental cues.^11^^,^^14^ Therefore, GFAP expression might be too instable for use as a reproducible prognostic marker. In this line, absence of GFAP-positive cells does not exclude the presence of enteric glia in intratumoral stroma. Of note, we showed that normal mucosa and adenoma tissue harbor S100B-positive enteric glia that are not immunoreactive for GFAP. More stable and broadly expressed enteric glial markers might be of interest to assess the prognostic value of enteric glial cells.

To evaluate GFAP immunoreactivity, we used a division of three different staining groups: low-density, high-density and negative staining for GFAP. This method was used because we observed a large variation in the amount of GFAP staining within the GFAP-positive patient samples. A full quantitative analysis was not applied as it would be necessary to assess the entire tumor rather than a single thin section. This would require evaluating multiple sections throughout the tumor or applying tissue-clearing and whole-tumor imaging techniques, which would allow for quantification of both the tumor area and the number of immunoreactive glial cells.^41^^,^^42^ Since our aim was to explore the potential of this staining method in a prognostic setting, such approaches proved too labor-intensive and would have required an impractical amount of tumor tissue. The applied semi-quantitative assessment strategy offered a practical and reproducible balance between objectivity and feasibility for a clinical research context. We did not evaluate staining intensity, as intensity differences were not representative for differences in GFAP expression on the biological level, based on corresponding overall slide and plexus intensity, and because staining intensity can be heavily affected by technical factors such as fixation and storage conditions.^43^^,^^44^ Studies that previously analyzed enteric glia in CRC used varying approaches to evaluate marker expression. Defining the total area of staining as percentage of the total area of tissue was used before but this method mostly evaluated plexus regions in or close to the tumor.^24^^,^^25^ In the present study, we evaluated the staining of enteric glia specifically in the tumor stroma and therefore excluded plexus structures from the analysis. The only prognostic study on enteric glial cells also using the GFAP marker divided patients over two equal groups namely, GFAP/S100B-high and -low, above or under the median.^23^ However, dividing the whole population in two equal size groups does not offer a clear representation of the observed staining differences throughout all tissue slides in our cohorts.

To conclude, our data propose glial reactivity in the CRC context as enteric glia upregulate GFAP expression in a cancerous environment. There seems to be no clear association between GFAP-positive enteric glia and CRC patient characteristics. Although GFAP appears to harbor some prognostic potential in our study cohort, this was not seen in the in-cohort validation. As GFAP expression is highly dynamic and phenotype-dependent, this enteric glial marker might not be the best candidate for a prognostic biomarker. Future studies should focus on other more stable glial markers or a combination of multiple markers for CRC prognosis. Furthermore, the biological effects of enteric glial plasticity on CRC progression have to be studied further.

## Supplementary Material

nlaf067_Supplementary_Data

## Data Availability

All data supporting the findings in this study are available in the article and supplementary information files and from the corresponding author upon reasonable request.
