# Supplementary material for: Clinicopathological characterization of enteric glia in colorectal cancer: Insights from a population-based cohort
Source: J Neuropathol Exp Neurol. 2025 Jun 28;84(10):892–901. doi: 10.1093/jnen/nlaf067 (PMC12456883; doi:10.1093/jnen/nlaf067)
Supplement: nlaf067_Supplementary_Data [file nlaf067_supplementary_data.zip › Supplemental Table.docx]

**Table S1.** Patient characteristics for cases where GFAP staining was not successful

| **Patient Demographics** | **Study cohort, n (%)** | **In-cohort validation, n (%)** |
| --- | --- | --- |
| **Total** | **89 (19.9)** | **64 (19.8)** |
| **Sex** |  |  |
| Male | 52 (58.4) | 33 (51.6) |
| Female | 37 (41.6) | 31 (48.4) |
| **Age at diagnosis (years)** |  |  |
| Mean Age (± SD) | 67.4 (4.3) | 73.9 (5.6) |
| **Cancer stage (TNM)** |  |  |
| Stage I | 26 (31.7) | 13 (21.3) |
| Stage II | 20 (24.4) | 22 (36.1) |
| Stage III | 20 (24.4) | 16 (26.2) |
| Stage IV | 16 (19.5) | 10 (16.4) |
| **Localization** |  |  |
| Colon | 46 (51.7) | 41 (64.1) |
| Rectosigmoid | 15 (16.8) | 8 (12.5) |
| Rectum | 28 (31.5) | 15 (23.4) |
| **Differentiation grade** |  |  |
| Undifferentiated | 1 (1.4) | - |
| Poor | 7 (9.6) | 9 (15.5) |
| Moderate | 51 (69.9) | 40 (69.0) |
| Well | 14 (19.2) | 9 (15.5) |
| **CRC death** |  |  |
| Yes | 43 (48.3) | 34 (53.1) |
| No | 46 (51.7) | 30 (46.9) |
| **Survival (years)** |  |  |
| Median (± SD) | 4.9 (5.1) | 2.8 (5.5) |

CRC = colorectal cancer, n = number of patients, SD = standard deviation, TNM = tumor-lymph node-metastasis
